# Antitumor effect of alcohol extract of *Isaria felina* on H22 hepatocellular carcinoma *in vitro* and *vivo*

**DOI:** 10.3389/fonc.2026.1789267

**Published:** 2026-08-12

**Authors:** Xiaoyan Li, Binxuan Wang, Guoyan Yu, Xiaochen Qiao, Ziyang Huang, Yuxin Yang, Junjun Li, Jinhua Xu, Liting Bai, Huimin Li, Xinyu Song, Lixia Chen, Lili Zhao, Yongming Yang, Yong Zhang, Jinmiao Tian, Xihua Yang

**Affiliations:** 1 Intensive Care Unit, Shanxi Province Cancer Hospital/Shanxi Hospital Affiliated to Cancer Hospital, Chinese Academy of Medical Sciences/Cancer Hospital Affiliated to Shanxi Medical University, Taiyuan, Shanxi, China; 2 Laboratory Animal Center, Shanxi Province Cancer Hospital/Shanxi Hospital Affiliated to Cancer Hospital, Chinese Academy of Medical Sciences/Cancer Hospital Affiliated to Shanxi Medical University, Taiyuan, Shanxi, China; 3 Department of Nutrition and Food Hygiene, School of Public Health, Shanxi Medical University, Taiyuan, Shanxi, China; 4 The Second Hospital of Shanxi Medical University/The Second Clinical Medical College of Shanxi Medical University/Shanxi Red Cross Hospital, Taiyuan, Shanxi, China; 5 School of Pharmacy, Shanxi Medical University, Taiyuan, Shanxi, China; 6 Endoscopy Center, Shanxi Province Cancer Hospital/Shanxi Hospital Affiliated to Cancer Hospital, Chinese Academy of Medical Sciences/Cancer Hospital Affiliated to Shanxi Medical University, Taiyuan, Shanxi, China

**Keywords:** alcohol extract, apoptosis, hepatocellular carcinoma, immunity, *Isaria felina*

## Abstract

**Background:**

Cordyceps sinensis is a well-known traditional Chinese medicine with antitumor activity. *Isaria felina* (IF) is a novel fungus isolated from the fruiting bodies of Cordyceps sinensis. IFA was prepared by fermentation and ethanol extraction. This study investigated the antitumor effects of the ethanol extract of IF (IFA) on hepatocellular carcinoma (HCC) using H22 hepatocellular carcinoma cells and a mouse xenograft model.

**Methods:**

*In vitro*, CCK-8 assay was used to assess cell viability. Annexin V-PE/7AAD flow cytometry, RT-PCR, and Western blot were performed to detect apoptosis and related pathways. *In vivo*, H22 tumor-bearing mice were treated with IFA by gavage. TUNEL staining, immunohistochemistry, blood routine, serum biochemistry, and ELISA were applied to evaluate tumor apoptosis, liver function parameters, and immune parameters.

**Results:**

IFA dose-dependently inhibited H22 cell proliferation. Flow cytometry showed that IFA induced late-stage apoptosis. RT-PCR and Western blot revealed upregulation of P53, Bax, Cyt C, and Caspase-3, and downregulation of Bcl-2, indicating activation of the P53/Bax/Bcl-2/Cyt C/Caspase-3 pathway. *In vivo*, IFA suppressed tumor growth, increased TUNEL-positive apoptotic cells, and enhanced serum TNF-α while reducing TGF-β, without impairing liver or kidney function.

**Conclusions:**

IFA inhibits HCC growth by inducing mitochondrial apoptosis via the P53 pathway and by modulating host immunity, highlighting its potential as a natural therapeutic agent for HCC.

## 1 Introduction

The global burden of liver cancer continues to rise. According to the GLOBOCAN 2022 estimates published by the International Agency for Research on Cancer (IARC) in April 2024, there were approximately 866,000 new cases and 759,000 deaths from liver cancer worldwide, making it the sixth most common cancer and the third leading cause of cancer-related death globally (1, 2). Hepatocellular carcinoma (HCC) accounts for 75%-85% of primary liver cancers and is the predominant histological type (3). The prognosis of HCC remains poor, largely because the disease is often diagnosed at an advanced stage when curative surgical options are no longer available (4).

Despite the availability of various systemic therapeutic agents for advanced HCC, their clinical utility is often constrained by limited efficacy, acquired drug resistance, and significant toxicity (5, 6). Moreover, conventional chemotherapeutic agents are frequently associated with severe side effects such as myelosuppression and hepatotoxicity, which further limit their long-term application (7). In this context, searching for natural products with potent antitumor activity and favorable safety profiles remains an important direction in anticancer drug development (6).

Cordyceps sinensis is a treasured traditional Chinese medicine, recorded in Chinese materia medica for its effects of “tonifying the lung and kidney, stopping bleeding and resolving phlegm.” Modern pharmacological studies have confirmed that Cordyceps sinensis contains nucleosides, polysaccharides, sterols, peptides, and other active components, exhibiting anti-inflammatory, antioxidant, antitumor, and immunomodulatory activities (8, 9). Among these, cordycepin, the most characteristic nucleoside component, has been shown to induce apoptosis in HCC cells through both extrinsic and intrinsic pathways, involving Bcl-2 family protein-mediated regulation of mitochondrial membrane potential, release of cytochrome c, and activation of the caspase cascade (10, 11). Zhou et al. (2017) further demonstrated that cordycepin can exert anti-HCC effects by modulating multiple pro-apoptotic and anti-apoptotic pathways (11). More recently, cordycepin has been found to regulate the Hippo signaling pathway in HCC, with YAP1 participating in the regulation of Bax and Bcl-2 expression (12). However, wild Cordyceps sinensis is scarce and expensive, making large-scale application difficult. Consequently, isolating strains from Cordyceps sinensis and conducting artificial fermentation culture has become a major approach to substituting natural cordyceps resources (13, 14).

*Isaria felina* (IF) is a fungal strain isolated from the fruiting bodies of Cordyceps sinensis by our research group, identified by the Institute of Microbiology, Chinese Academy of Sciences, and deposited at the China General Microbiological Culture Collection Center (CGMCC NO.0706). In previous work, we completed the optimization of fermentation conditions and the analysis of active components, finding that the ethanol-soluble fraction of IF (IFA) is rich in nucleoside compounds. Yang et al. (2023) reported that selenium-enriched IF could enhance *in vivo* antitumor effects on H22 hepatoma by downregulating VEGF expression (15); Shi et al. (2024) showed that IF combined with cyclophosphamide exerted additive antitumor effects in H22 tumor-bearing mice and alleviated cyclophosphamide-induced immunosuppression (16). In addition, Liu et al. systematically reviewed the antitumor components and mechanisms of Cordyceps, pointing out that nucleoside compounds are among the major active antitumor constituents (17). However, the systematic pharmacodynamic evaluation and molecular mechanisms of IFA against HCC have not yet been reported. Based on the above understanding, the present study aimed to investigate the inhibitory effects of IFA on H22 mouse hepatocellular carcinoma cells both *in vitro* and *in vivo*, and explored the involvement of the P53/Bax/Bcl-2/Cyt C/Caspase-3 pathway by examining the expression levels of key proteins and genes, in order to provide a new candidate agent for HCC therapy.

## 2 Materials and methods

### 2.1 Cell lines and culture

The H22 mouse hepatoma cells were generously provided by the Experimental Animal Center of Hebei Medical University and are stored at the Animal Experiment Center of Shanxi Cancer Hospital. Cells were recovered and cultured in RPMI-1640 medium supplemented with 10% fetal bovine serum, 100 U/mL penicillin, and 100 μg/mL streptomycin at 37 °C in a 5% CO_2_ incubator. When cell confluence reached 80%-90%, the cell suspension was collected, centrifuged at 1000 r/min for 5 min, and the supernatant was discarded. Fresh medium was added to resuspend the cells, and subculture was performed at an appropriate ratio.

### 2.2 Preparation of IFA

The IF strain (CGMCC NO.0706) was inoculated into liquid fermentation medium containing 2% glucose, 1% peptone, 0.5% yeast extract, 0.1% KH_2_PO_4_, and 0.05% MgSO_4_·7H_2_O at pH 6.0, and cultured at 25 °C with shaking at 150 r/min for 7 days. After fermentation, the mycelium was collected, lyophilized, and ground into powder. The powder was extracted with 50% ethanol at a solid-to-liquid ratio of 1:10 at 60 °C for 2 hours, repeated three times. The combined extracts were concentrated under reduced pressure and lyophilized to obtain IFA powder. Before use, the powder was dissolved in saline at the desired concentration.

### 2.3 Animals and model establishment

Specific pathogen-free (SPF) female Kunming mice, aged 6 weeks and weighing 18-22 g, were provided by the Experimental Animal Center of Shanxi Cancer Hospital [License No. SCXK (Jin) 2022-0002]. Mice were housed in a barrier environment at 22-24 °C, 40%-60% relative humidity, with a 12-h light/dark cycle. After one week of acclimatization, during which food and water were provided ad libitum, the experiment began. This study was approved by the Animal Welfare and Ethics Committee of Shanxi Cancer Hospital (Approval No. 20220806).

Mice that had been intraperitoneally inoculated with H22 cells 7 days earlier were selected. Under sterile conditions, ascites <font color=red>was</font> aspirated, washed twice with phosphate-buffered saline (PBS), and counted. The cell concentration was adjusted to 1×10^6^ cells/mL, and 0.1 mL of the cell suspension was subcutaneously inoculated into the right axilla of each mouse. Nodule formation at the inoculation site within approximately 6 days indicated successful modeling.

### 2.4 Grouping and drug administration

*In vitro* experiment: H22 cells were divided into three groups: control (Ctrl), IFA low-dose (IFA-L, 4 mg/mL), and IFA high-dose (IFA-H, 8 mg/mL), with treatment for 24 hours. Doses were selected based on the IC_50_ results.

*In vivo* experiment: Thirty-six mice were randomly divided into six groups (n=6 each): normal control (NC, no inoculation, saline gavage), model control (MC, H22 inoculation, saline gavage), positive control (CTX, H22 inoculation, cyclophosphamide 25 mg/kg intraperitoneal injection), IFA low-dose (37.5 mg/kg gavage), IFA medium-dose (75 mg/kg gavage), and IFA high-dose (150 mg/kg gavage). Drug administration began on the second day after inoculation and continued for 10 consecutive days.

### 2.5 Cell viability assay

H22 cells were seeded in 96-well plates at 5×10³ cells/well and treated with IFA at final concentrations of 0, 1, 2, 4, 8, and 16 mg/mL, with three replicate wells per group. After 24 hours of treatment, 10 μL of CCK-8 solution was added to each well and incubated at 37 °C for 2 hours. Absorbance was measured at 450 nm (reference 630 nm) using a microplate reader. Cell morphology was observed and photographed under an inverted microscope (scale bar: 50 μm, magnification: 200×).

### 2.6 Flow cytometric detection of apoptosis

H22 cells were seeded in 6-well plates at 1×10^4^ cells/mL and treated with IFA for 24 hours. Cells were collected, washed with PBS, and resuspended in 100 μL of binding buffer. Then 5 μL of Annexin V-PE was added and incubated in the dark at room temperature for 5 minutes, followed by the addition of 10 μL of 7-AAD and 400 μL of PBS. After mixing, the samples were immediately analyzed by flow cytometry.

### 2.7 Real-time quantitative PCR

Total RNA was extracted from cells or tumor tissues using the Trizol method and reverse-transcribed into cDNA. GAPDH was used as the internal control, and amplification was performed using the SYBR Green method. Primer sequences are provided in the **Supplementary Materials**. Relative expression was calculated using the 2^^-^ΔΔ^*^Ct^* method. Primer sequences used in this study are all shown in **Supplementary Table 1**.

### 2.8 Western blot

Total protein was extracted using RIPA lysis buffer and quantified by the BCA method. Thirty micrograms of protein were separated by SDS-PAGE and transferred to PVDF membranes. After blocking with 5% skim milk, the membranes were incubated with primary antibodies against P53, Bax, Bcl-2, Cyt C, Caspase-3, Cleaved Caspase-3, and β-actin overnight at 4 °C. The next day, after washing, the membranes were incubated with HRP-conjugated secondary antibodies for 2 hours at room temperature, and signals were detected by ECL. Band intensities were analyzed using Image Pro Plus 6.0. (Full-length uncropped membrane images could not be retrieved because the membranes underwent stripping and reprobing during the experiment; the cropped band images are provided in **Supplementary Figure 1**).

### 2.9 TUNEL staining

Paraffin sections of tumor tissues were deparaffinized, rehydrated, and subjected to antigen retrieval in sodium citrate buffer using microwave treatment. Endogenous peroxidase was blocked with 3% H_2_O_2_. The sections were then incubated with TUNEL reaction mixture, and nuclei were counterstained with DAPI. Images were observed and captured under a laser confocal microscope (magnification: 200×). Five random fields per section were selected, and the proportion of TUNEL-positive cells was counted.

### 2.10 Blood collection, routine and serum biochemistry

On the day after the last administration, mice were anesthetized with intraperitoneal sodium pentobarbital, and blood was collected by enucleation of the eyeballs. One portion of the blood was collected into EDTA-K_2_ anticoagulant tubes for complete blood count analysis (white blood cells, red blood cells, lymphocytes) using an automated hematology analyzer. Another portion was allowed to stand at room temperature for 30 minutes, then centrifuged at 3000 r/min for 15 minutes to separate serum, which was used for the detection of urea nitrogen, creatinine, ALT, and AST using an automated biochemical analyzer.

### 2.11 ELISA

Serum samples were centrifuged at a speed of 3000 revolutions per minute for a duration of 15 minutes to obtain serum samples. The concentrations of TGF-β and TNF-α in the serum were then determined using ELISA kits (Fanke, Shanghai, China) on a microplate reader (Tecan, Männedorf, Switzerland), adhering strictly to the manufacturer’s guidelines.

### 2.12 Statistical analysis

Statistical analysis was conducted using SPSS 26.0 software. Data are presented as mean ± standard deviation ( $\bar{x}$ ± SD). One-way ANOVA was used to analyze the overall differences. For pairwise comparisons, the LSD method was employed when the variances between the two groups were equal, whereas the Dunnett method was used when the variances were unequal. A P-value < 0.05 was considered statistically significant.

## 3 Results

### 3.1 IFA induces late apoptosis in H22 cells

**Figure 1:**
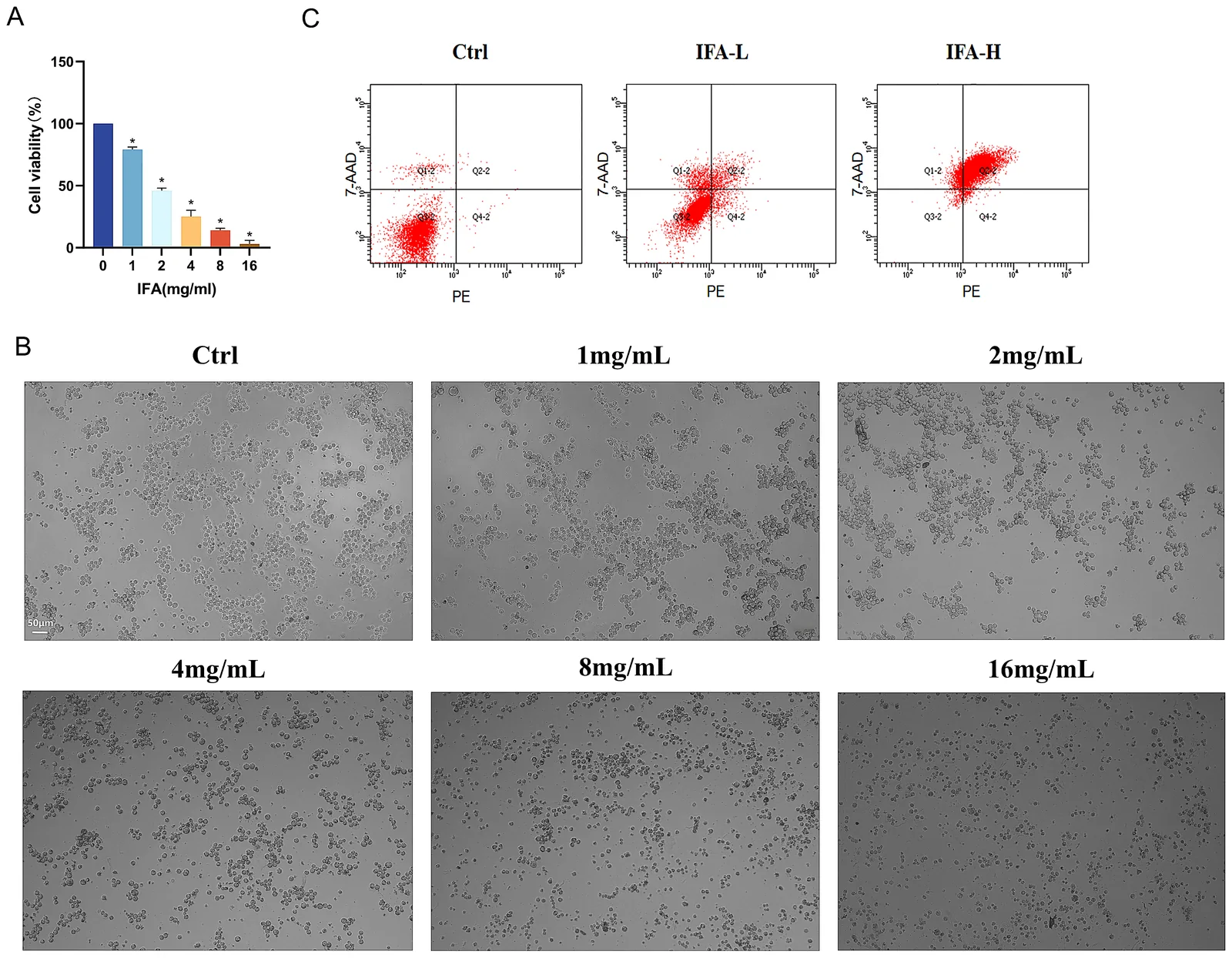
Effects of IFA on cell viability, apoptosis, and morphology. **(A)** Cell viability of H22 cells treated with serial concentrations of IFA (0, 1, 2, 4, 8, 16 mg/mL) for 24 h. Nonlinear curve fitting was conducted to calculate the IC_50_ value of IFA (6.2 mg/mL). **(B)** Morphological microscopic images of H22 cells treated with various concentrations of IFA (0, 1, 2, 4, 8, 16 mg/mL). Scale bar 50 μm, magnification 200×. **(C)** Flow cytometry analysis of cell apoptosis in control (Ctrl), low-dose IFA (IFA-L), and high-dose IFA (IFA-H) groups. Data are shown as mean ± SD, n=3. *P < 0.05 compared with the Ctrl group.

Previous research conducted by our research group has demonstrated that IF has an inhibitory effect on H22 tumor-bearing mice. Furthermore, it was determined that the alcohol-soluble substances of IF contain a large amount of nucleosides. Numerous studies have indicated that nucleosides are the main active components of fermented Cordyceps sinensis with antitumor properties. Therefore, we extracted IFA from IF using ethanol to investigate whether IFA also exhibits inhibitory effects on liver cancer in mice. H22 cells were treated with different concentrations of IFA solution (1, 2, 4, 8, 16 mg/mL) for 24 hours, and cell viability was assessed using the CCK-8 method. The results showed that compared to the control group (Ctrl), the survival rate of H22 cells decreased after IFA solution intervention in a concentration-dependent manner, as presented in **Supplementary Table 2**; **Figure 1A**. Nonlinear curve fitting based on cell viability data was performed to calculate the half-maximal inhibitory concentration (IC_50_) of IFA against H22 cells, which was 6.2 mg/mL. Accordingly, 4 mg/mL and 8 mg/mL were selected as low-dose (IFA-L) and high-dose (IFA-H) concentrations for subsequent apoptosis and molecular mechanism assays. During the process of using the CCK-8 method to detect the effect of IFA on tumor cell viability, we observed that the cells in the control group (Ctrl) appeared transparent and glossy. However, the morphology of H22 cells after IFA intervention underwent significant changes, becoming dull and deformed, with reduced cell volume and even fragmentation (**Figure 1B**). This demonstrates that IFA has an inhibitory effect on the growth of H22 cells.

Based on the morphological changes of H22 cells after IFA treatment and previous studies, we hypothesized that the inhibitory effect of IFA on H22 hepatoma cells is related to the induction of apoptosis. To investigate whether IFA can induce apoptosis of H22 cells, H22 cells were divided into three groups: Ctrl group, IFA-L group and IFA-H group. The apoptosis rate was measured using flow cytometry, as shown in **Figure 1C**; **Supplementary Table 3**; the I, II, III and IV quadrants represent necrotic cells, late apoptotic cells, normal cells and early apoptotic cells, respectively. The results showed that IFA could induce apoptosis of H22 cells, and the percentage of apoptotic cells increased with the increase of concentration. The early apoptosis rate of the Ctrl group, IFA-L group and IFA-H group was 0.44%, 3.03% and 3.51%, respectively, and the late apoptosis rate was 0.57%, 6.65% and 80.65%, respectively. The early apoptosis rate of the IFA-L group and IFA-H group was significantly higher than that of the Ctrl group (P < 0.05), and the late apoptosis rate of the IFA-L group and IFA-H group was also significantly higher than that of the Ctrl group (P < 0.05). These results suggest that IFA can induce apoptosis in H22 cells, especially late-stage apoptosis.

### 3.2 IFA induces apoptosis in H22 cells through the p53-mediated mitochondrial apoptosis pathway

**Figure 2:**
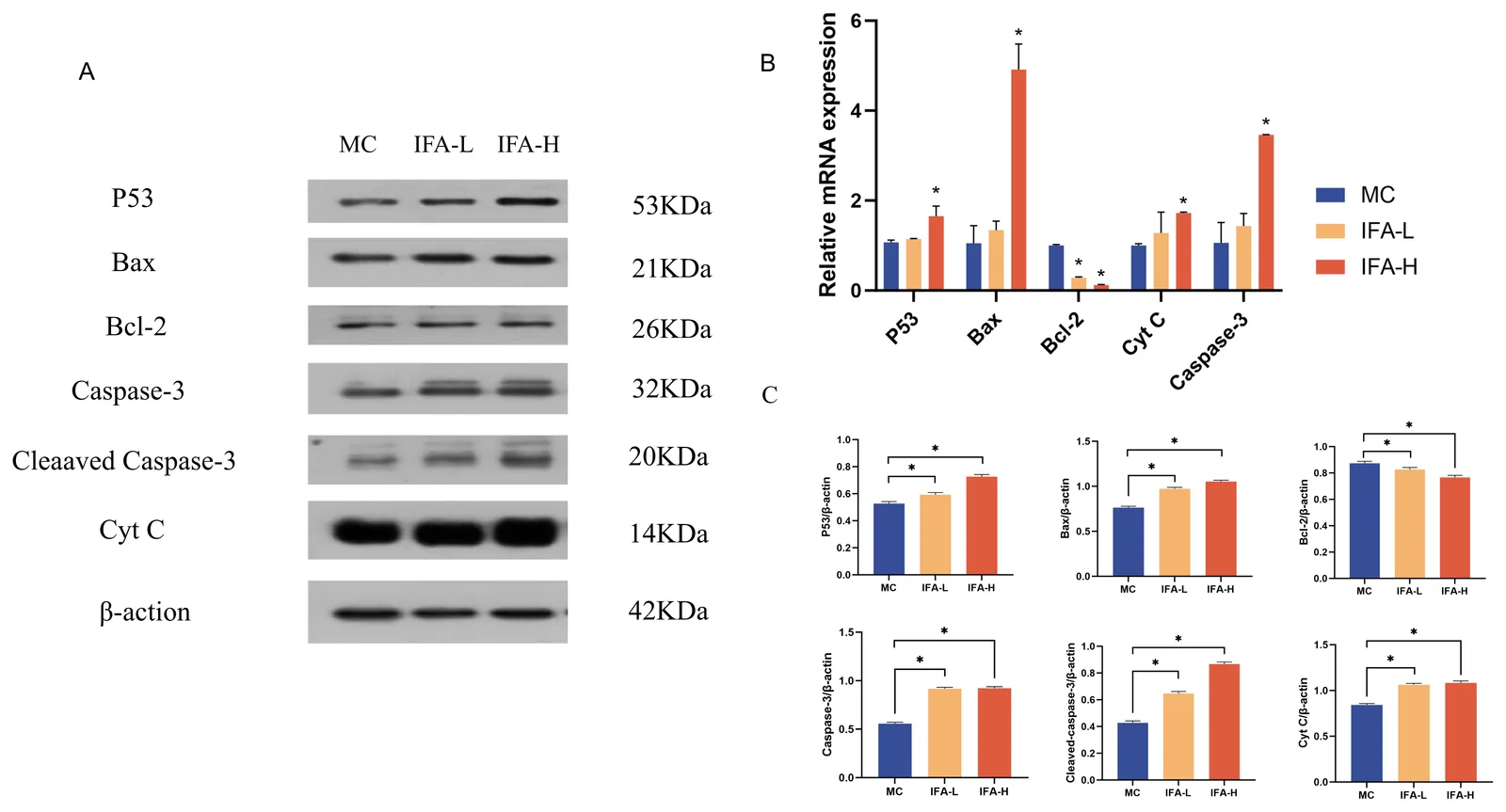
Effects of IFA on apoptosis-related protein and mRNA expression. **(A)** Western blot analysis of P53, Bax, Bcl - 2, Caspase - 3, Cleaved Caspase - 3, Cyt C, and β - actin protein levels in MC, IFA - L, and IFA - H groups. **(B)** Relative mRNA expression levels of P53, Bax, Bcl - 2, Cyt C, and Caspase - 3 in MC, IFA - L, and IFA - H groups. **(C)** Quantitative analysis of P53, Bax, Bcl - 2, Caspase - 3, Cleaved Caspase - 3, and Cyt C protein expression normalized to β - actin in MC, IFA - L, and IFA - H groups. Data are shown as mean ± SD,n=3.*P < 0.05 compared with the Ctrl group.

To ascertain the changes in apoptosis-related mRNA expression in H22 cells following IFA intervention, we further employed real-time fluorescent quantitative PCR to detect the expression levels of apoptosis-related mRNAs in various cell groups. Compared with the Ctrl group, the relative expression levels of *P53, Bax, Cyt C, and Caspase-3* mRNAs were upregulated in the IFA-H group, while the relative expression level of *Bcl-2* mRNA was downregulated as shown in **Figure 2A**. To examine the changes in apoptosis-related protein expression following IFA treatment, we performed Western blot analysis on the various cell groups. Following IFA intervention in H22 cells, the expression levels of P53, Bax, Cyt C, and Caspase-3 proteins were upregulated in both the IFA-L and IFA-H groups compared to the Ctrl group. Conversely, Bcl-2 expression was downregulated. These results are presented in **Figures 2B, C**.

### 3.3 IFA induces apoptosis in tumor tissues of H22 tumor-bearing mice

**Figure 3:**
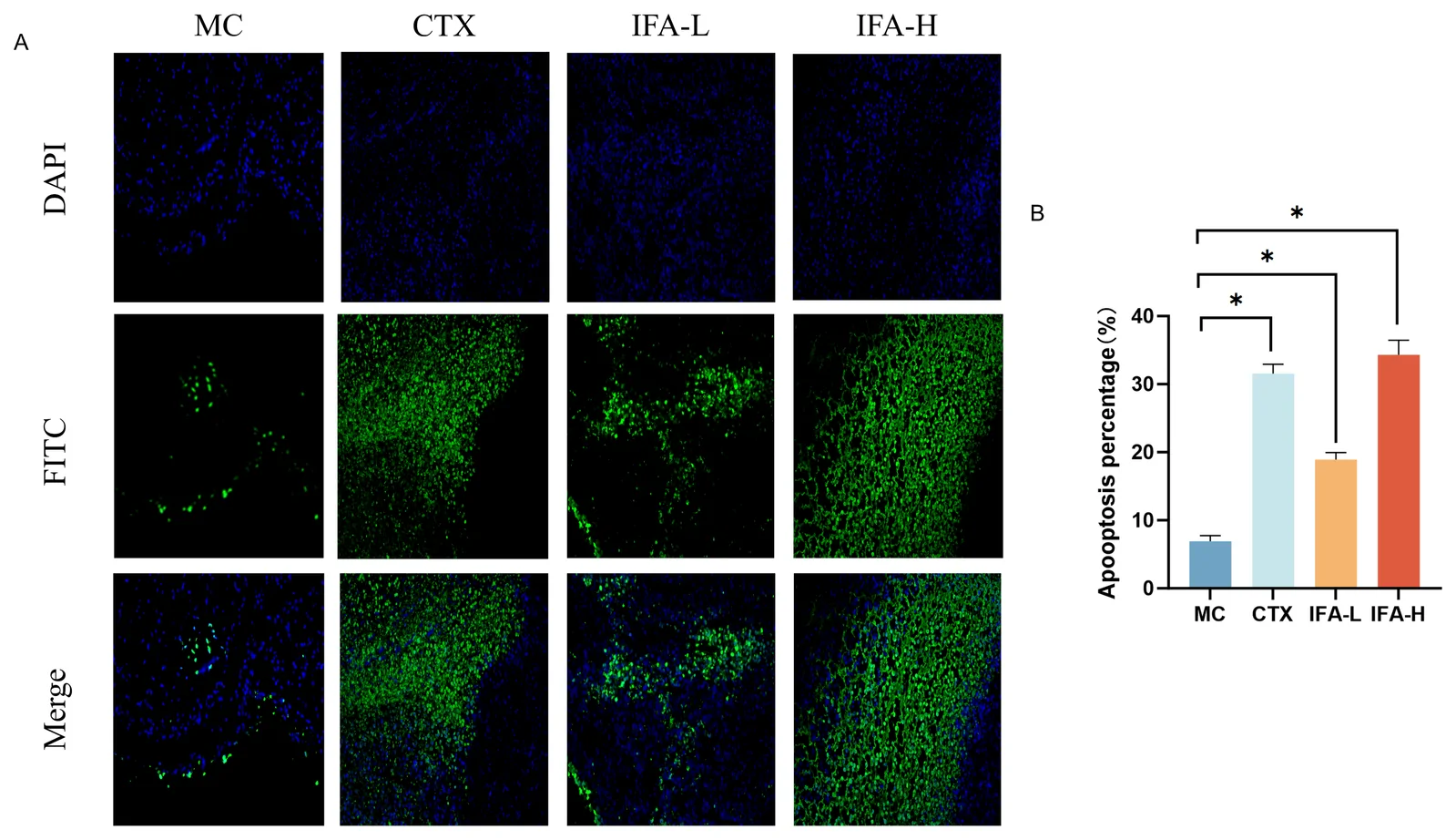
Effects of IFA on cell apoptosis detected by immunofluorescence staining. **(A)** Immunofluorescence TUNEL staining images of tumor tissues from MC, CTX, IFA-L, and IFA-H groups. Nuclei were stained with DAPI (blue), and apoptotic cells were stained with FITC (green). Merge images show the overlay of DAPI and FITC signals. Magnification 200×. **(B)** Quantitative statistical analysis of apoptotic cell percentage in MC, CTX, IFA-L, and IFA-H groups. MC, untreated tumor model mice; CTX, cyclophosphamide-treated tumor model mice. Data are shown as mean ± SD, n=3. *P < 0.05 compared with the MC group.

To verify whether IFA has an inhibitory effect on liver cancer *in vivo*, we established an H22 liver cancer mouse model. Apoptosis in tumor tissue cells of tumor-bearing mice from each group was detected using TUNEL staining, with the results shown in **Figure 3A**: Blue fluorescence represents all cells in the mouse tumor tissue sections, while green fluorescence indicates apoptotic cells in the tissue sections. Quantitative analysis using the formula [apoptotic index = (number of TUNEL-positive cells/total number of cells) × 100%] revealed that the proportion of apoptotic cells in the tumor tissue of the MC group was 6.48%. Compared with the MC group, the apoptotic indices of the CTX group (34.25%), IFA-L group (18.44%), IFA-M group (28.22%), and IFA-H group (37.87%) were all significantly increased (P < 0.05, **Figure 3B**). This demonstrates that IFA can promote apoptosis in tumor tissue cells *in vivo*.

### 3.4 IFA promotes the expression of apoptosis-related proteins in tumor tissues of H22 tumor-bearing mice

**Figure 4:**
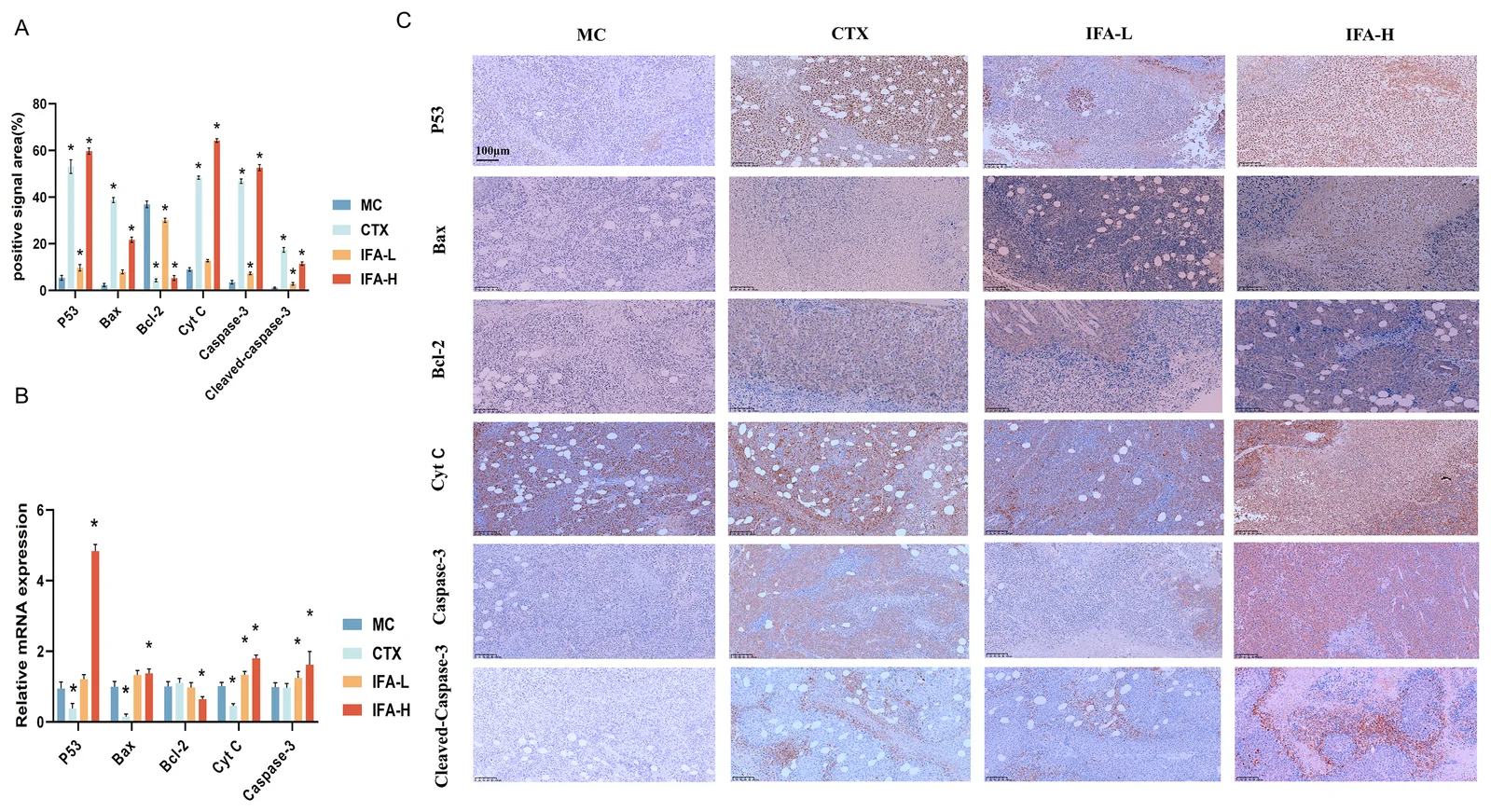
Effects of IFA on the expression of apoptosis - related proteins and mRNA in tissues. **(A)** Immunohistochemical staining images of P53, Bax, Bcl-2, Cyt C, Caspase-3, and Cleaved-Caspase-3 in MC, CTX, IFA-L, and IFA-H groups. Scale bar 100 μm, magnification 400×. **(B)** Relative mRNA expression levels of P53, Bax, Bcl-2, Cyt C, and Caspase-3 in MC, CTX, IFA-L, and IFA-H groups. **(C)** Quantitative analysis of the positive signal area of P53, Bax, Bcl-2, Cyt C, Caspase-3, and Cleaved-Caspase-3 in MC, CTX, IFA-L, and IFA-H groups. MC, untreated tumor model mice; CTX, cyclophosphamide-treated tumor model mice. Data are shown as mean ± SD, n=3. *P < 0.05 compared with the MC group.

To confirm that IFA can also promote tumor cell apoptosis *in vivo*, we used immunohistochemical methods to detect changes in the expression levels of apoptosis-related proteins in tumor tissues of tumor-bearing mice from each group. Compared with the MC group, the expression levels of P53 protein were upregulated in the CTX group and all IFA groups. The expression levels of Bax protein were upregulated in the CTX group, IFA-M group, and IFA-H group. The expression levels of Bcl-2 protein were downregulated in the CTX group, IFA-M group, and IFA-H group. The expression levels of Cyt C protein were upregulated in the CTX group, IFA-M group, and IFA-H group. The expression levels of Caspase-3 protein were upregulated in the CTX group and all IFA groups. The expression of Cleaved-caspase-3 was also elevated in the CTX group and all IFA groups, as shown in **Figures 4A, B**. We further employed real-time fluorescent quantitative PCR to detect the expression of apoptosis-related mRNAs in tumor tissues of tumor-bearing mice from each group. Relative to the MC group, the IFA-H group showed upregulated mRNA expression of P53, Bax, Cyt C, and Caspase-3, and downregulated expression of Bcl-2, as shown in **Figure 4C**. The trends in the expression of apoptosis-related proteins in tumor tissues were generally consistent with those in cells, indicating that the mechanism by which IFA promotes tumor cell apoptosis *in vivo* is also related to the P53-mediated mitochondrial apoptosis pathway.

### 3.5 IFA has no significant toxic side effects on H22 tumor-bearing mice

**Figure 5:**
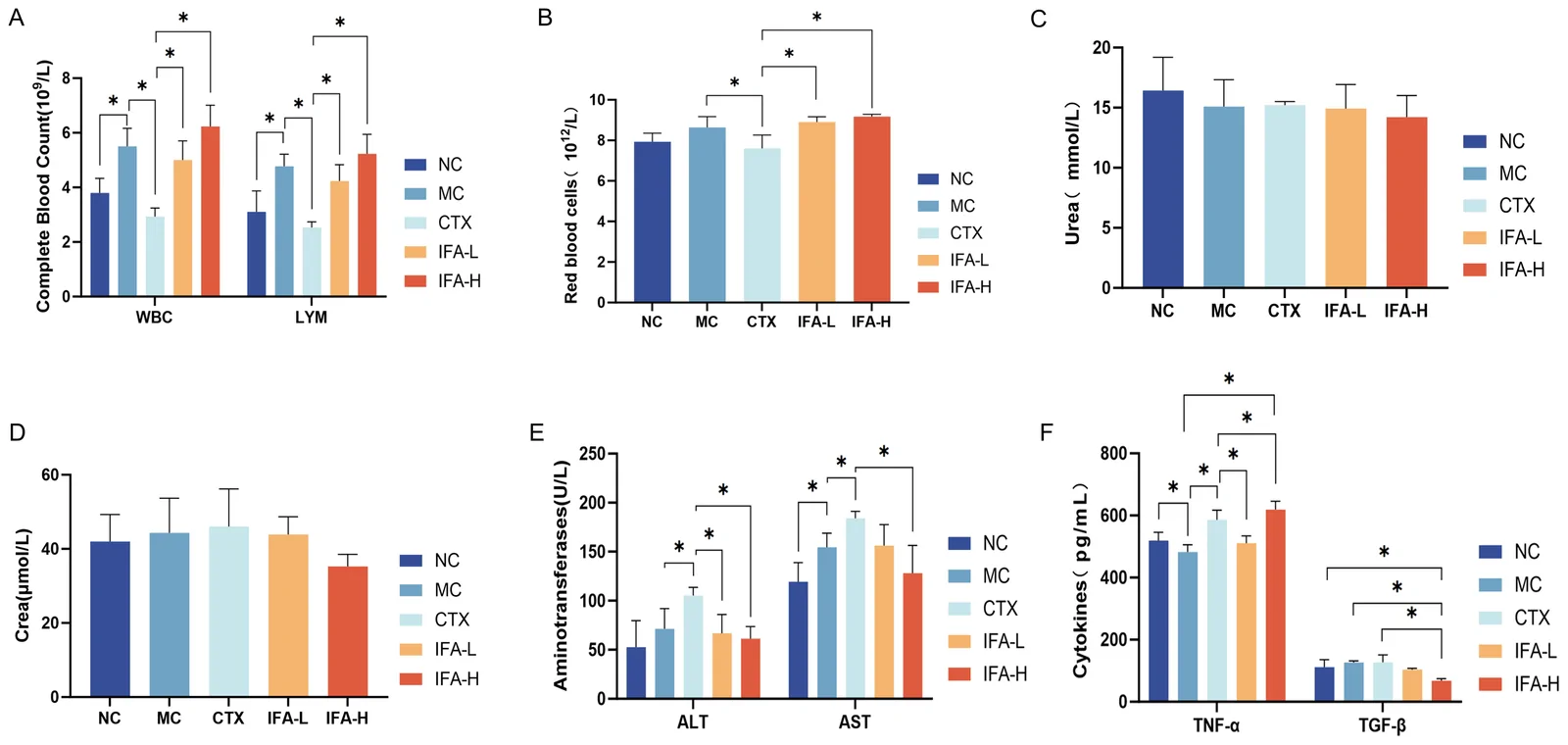
Effects of IFA on blood parameters and cytokine levels. **(A)** Complete blood count (WBC, white blood cell; LYM, lymphocyte) in NC, MC, CTX, IFA - L, and IFA - H groups. **(B)** Red blood cell count in NC, MC, CTX, IFA - L, and IFA - H groups. **(C)** Urea level in NC, MC, CTX, IFA - L, and IFA - H groups. **(D)** Creatinine (Crea) level in NC, MC, CTX, IFA - L, and IFA - H groups. **(E)** Aminotransferases (ALT, alanine aminotransferase; AST, aspartate aminotransferase) levels in NC, MC, CTX, IFA - L, and IFA - H groups. **(F)** Cytokines (TNF - α: tumor necrosis factor - α; TGF - β: transforming growth factor - β) levels in NC, MC, CTX, IFA - L, and IFA - H groups. NC, normal mice; MC, untreated tumor model mice; CTX, cyclophosphamide treated tumor model mice. Data are shown as mean ± SD, n=3. *P < 0.05.

The blood routine test results for mice in various groups are presented in **Figures 5A, B**; **Supplementary Table 4**. Compared with the NC group, the MC group exhibited increased levels of white blood cells (WBCs) and lymphocytes. In contrast, when compared with the MC group, the CTX group showed significant decreases in the counts of WBCs, red blood cells (RBCs), and lymphocytes. When compared with the CTX group, the IFA groups demonstrated significant increases in the counts of WBCs, RBCs, and lymphocytes. These findings suggest that CTX induces bone marrow suppression in mice, manifested prominently by decreased levels of WBCs, lymphocytes, and RBCs, whereas IFA does not exhibit notable bone marrow suppressive side effects. Using an automated blood chemistry analyzer, the levels of serum Urea, Crea, ALT and AST were measured in mice. The results are presented in **Figures 5C–F**; **Supplementary Tables 5**, **S6**. There were no significant differences in serum Urea and Crea levels among the various groups. Compared with the MC group, the CTX group showed a significant increase in serum ALT levels. When compared with the CTX group, the IFA groups exhibited significant decreases in serum ALT levels. Compared with the NC group, the MC group demonstrated a significant increase in serum AST levels. Similarly, when compared with the MC group, the CTX group showed a significant elevation in serum AST levels. In comparison to the CTX group, the medium and high-dose IFA groups exhibited significant decreases in serum AST levels. ALT and AST are sensitive indicators of liver function, and elevations in both enzymes suggest liver damage. These results demonstrate that CTX affects liver function in mice, whereas IFA does not have an adverse impact on liver function in mice.

## 4 Discussion

Cordyceps sinensis is a well-known traditional Chinese medicine with documented antitumor and immunomodulatory properties. Its active components, particularly cordycepin, have been extensively studied for their ability to induce apoptosis in various cancer cell lines through both extrinsic and intrinsic pathways (10, 11). The mitochondrial pathway, regulated by the Bcl-2 family of proteins, plays a central role in this process, as cordycepin has been shown to alter mitochondrial membrane potential, promote cytochrome c release, and activate the caspase cascade (11, 12). However, the high cost and scarcity of natural Cordyceps sinensis have limited its widespread application, prompting the exploration of fermented substitutes derived from isolated strains such as *Isaria felina* (13, 14). In the present study, we systematically evaluated the antitumor effects of the ethanol extract of *Isaria felina* (IFA) against H22 hepatocellular carcinoma both *in vitro* and *in vivo*. To our knowledge, this is the first report demonstrating that IFA exerts significant antitumor activity through induction of mitochondrial apoptosis and modulation of immune responses.

The *in vitro* experiments showed that IFA inhibited H22 cell proliferation in a concentration-dependent manner, with an IC_50_ value of 6.2 mg/mL. Although this value is higher than that reported for purified cordycepin, it is consistent with the expected activity of a crude extract containing a limited proportion of active components (10, 12). More importantly, IFA induced apoptosis in H22 cells predominantly at the late stage, with the high-dose group exhibiting an 80.65% late apoptosis rate. This predominance of late apoptosis is characteristic of mitochondrial pathway activation, as cells undergoing mitochondria-mediated apoptosis typically experience a prolonged execution phase following cytochrome c release and caspase activation (18, 19).

Regarding the potential mechanism, our results showed that IFA treatment elevated P53 expression, which was accompanied by increased Bax and decreased Bcl-2 expression, suggesting a shift in the Bax/Bcl-2 ratio toward pro-apoptotic signaling. These changes were associated with Cyt C release from mitochondria into the cytosol and subsequent Caspase-3 activation, consistent with the involvement of the mitochondrial apoptosis pathway (18–20). This mechanism was validated in both *in vitro* and *in vivo* experiments, demonstrating good reproducibility and stability of IFA’s effects. Similar modes of action have been reported for cordycepin and other Cordyceps-derived products, supporting the notion that inducing mitochondrial apoptosis may be a common mechanism among active substances originating from Cordyceps species (10–12).

The *in vivo* results further supported our *in vitro* findings. IFA administration significantly inhibited tumor growth in H22-bearing mice, and TUNEL staining confirmed a dose-dependent increase in apoptotic cells within tumor tissues. The TUNEL (terminal deoxynucleotidyl transferase-mediated dUTP nick end-labeling) method is a widely accepted technique for detecting DNA fragmentation, a hallmark of apoptosis, in tissue sections (21, 22). Immunohistochemical analysis revealed corresponding changes in apoptosis-related proteins—upregulation of P53, Bax, Cyt C, and Cleaved Caspase-3, and downregulation of Bcl-2—in tumor tissues from IFA-treated mice, corroborating the *in vitro* mechanistic findings.

It is noteworthy that the apoptotic index in the IFA-H group (37.87%) was comparable to that of the CTX group (34.25%), indicating that IFA can achieve pro-apoptotic effects similar to those of the conventional chemotherapeutic agent cyclophosphamide at appropriate doses. However, the safety profiles of the two agents were markedly different. CTX caused significant decreases in white blood cells, red blood cells, and lymphocytes, as well as elevations in ALT and AST, consistent with its well-documented bone marrow suppression and hepatotoxicity (23–25). In contrast, IFA treatment did not induce these hematological or hepatic abnormalities. Cyclophosphamide was selected as the positive control in this study because it is a well-established alkylating agent with known antitumor activity against H22 hepatoma, and its myelosuppressive and hepatotoxic side effects are well-characterized, making it an appropriate reference for comparing the safety profile of IFA. Although CTX is not a first-line agent for HCC in current clinical practice, its use as a reference compound in preclinical studies is widely accepted for validating experimental systems and benchmarking new drug candidates.

The immunomodulatory effects of IFA also deserve attention. In this study, IFA elevated serum TNF-α levels while reducing TGF-β levels. TNF-α is a pro-inflammatory cytokine with direct antitumor activity, whereas TGF-β in the HCC microenvironment predominantly exerts immunosuppressive and pro-tumorigenic effects (26, 27). The regulatory effects of natural products on immune cell function and cytokine networks have become a research hotspot in recent years (28–30). Multiple studies have confirmed that naturally derived polysaccharides, flavonoids, and alkaloids can enhance antitumor immune responses by modulating regulatory immune cell function and cytokine secretion (31, 32). The inverse regulation of TNF-α and TGF-β by IFA suggests that it may synergistically enhance its direct pro-apoptotic effect by improving the tumor immune microenvironment. This dual mechanism of “direct killing + immune activation” parallels the current strategy of combining targeted agents with immune checkpoint inhibitors in HCC therapy.

Several limitations of this study should be acknowledged. First, the chemical composition of IFA has not been fully elucidated. Although HPLC analysis indicated that it is rich in nucleosides, the specific active monomers and their synergistic effects require identification through LC-MS/MS combined with bioactivity-guided fractionation (32, 33). Second, the subcutaneous transplantation model used in this study does not fully recapitulate the growth microenvironment of HCC in the liver; future studies should employ orthotopic inoculation models for validation (33, 34). Third, the administration period was only 10 days, which is relatively short for evaluating long-term safety and drug resistance (35, 36). Fourth, upstream signaling molecules of the mitochondrial pathway, such as the phosphorylation status of P53 and the involvement of PI3K/Akt and MAPK pathways, warrant further investigation (37–40).

## 5 Conclusion

The ethanol extract of *Isaria felina* (IFA) concentration-dependently inhibited H22 cell proliferation and induced late-stage apoptosis *in vitro*, and suppressed tumor growth in H22-bearing mice *in vivo.* Mechanistically, IFA activated the P53/Bax/Bcl-2/Cyt C/Caspase-3 mitochondrial apoptosis pathway and modulated the TNF-α/TGF-β immune balance. At effective doses, IFA did not cause significant hepatorenal or hematopoietic toxicity, demonstrating a favorable safety profile. Collectively, these results suggest that IFA has potential for development as a candidate therapeutic agent for hepatocellular carcinoma.

## Data Availability

The datasets presented in this study can be found in online repositories. The names of the repository/repositories and accession number(s) can be found in the article/**Supplementary Material**.
