# Supplementary material for: Antitumor effect of alcohol extract of Isaria felina on H22 hepatocellular carcinoma in vitro and vivo
Source: Front Oncol. 2026 Aug 12;16:1789267. doi: 10.3389/fonc.2026.1789267 (PMC13506430; doi:10.3389/fonc.2026.1789267)
Supplement: Supplementary file 1 [file SupplementaryFile1.pdf]

Supplementary Table S1 Primer sequences

Gene Name	Forward	Reverse
P53	CCCTCTGAGCCAGGAGACATT	CCCAGGTGGAAGCCATAGTTG
Bax	GCCTTTTTGCTACAGGGTTTCAT	TATTGCTGTCCAGTTCATCTCCA
Bcl-2	TGACTTCTCTCGTCGCTACCGT	CCTGAAGAGTTCCTCCACCACC
Cyt C	5-AGGTGCCCGACTTCTCTGACTATC-3	5-CCGCATAAGCAACACCCACAGTAG-3
Caspase-3	GTCTGACTGGAAAGCCGAAAC	GACTGGATGAACCACGACCC
GADPH	5-ACCCCAGCAAGGACACTGAGCAAG-3	5-GGCCCCTCCTGTTATTATGGGGGT-3

Supplementary Figure 1 The cropped strip image

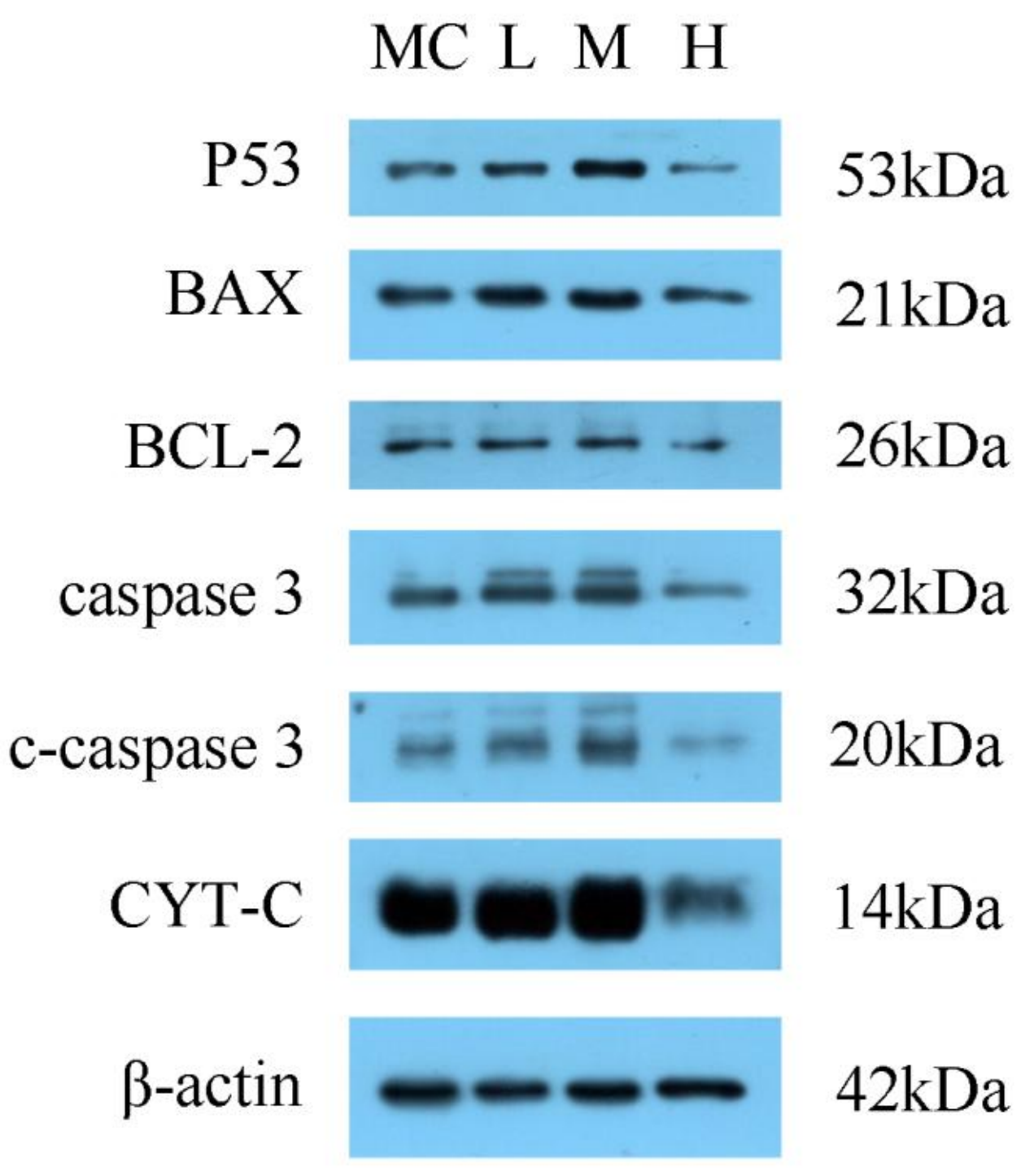

Supplementary Table S2 The impact of different concentrations of IFA on the survival rate of H22 cells（ $\bar{x} \pm SD$, n=3）

Groups	Cell viability(%)
Ctrl	100.00±0.00

IFA1 mg/mL	79.14±2.06*
IFA2 mg/mL	46.07±1.98*
IFA 4 mg/mL	25.04±5.16*
IFA 8 mg/mL	14.06±1.67*
IFA 16 mg/mL	3.03±2.85*

Supplementary Table S3 Effect of IFA H22 on apoptosis of hepatoma cells（ $\bar{x}$± SD, n=3）

Groups	Necrotic cells（%）	Late apoptosis（%）	Normal cells（%）	Early apoptosis（%）
Ctrl	2.79±0.94	0.57±0.23	96.20±1.07	0.44±0.30
IFA-L	6.04±0.87*	6.65±1.99*	84.28±1.94*	3.03±0.91*
IFA-H	11.37±1.10*	80.65±4.05*	4.47±2.18*	3.51±1.72*

Supplementary Table S4 Blood Routine Parameters of Mice in Each Group（ $\bar{x}$ ± SD）

Group	Number	White Blood Cells (10^9 /L)	Red Blood Cells (10^{12}/L)	Lymphocytes (10^9 /L)
NC	6	3.80±0.53	7.93±0.42	3.10±0.77
MC	6	5.50±0.66#	8.64±0.53	4.77±0.45#
CTX	6	2.93±0.31*	7.60±0.66*	2.53±0.21*
IFA-L	6	5.00±0.70△	8.90±0.26△	4.23±0.60△
IFA-M	6	4.37±0.25*△	8.93±0.42△	3.80±0.26*△
IFA-H	6	6.23±0.78△	9.17±0.12△	5.23±0.71△

Note: Compared with the NC group, #P<0.05; Compared with the MC group, *P < 0.05; Compared with the CTX group, △P<0.05.

Supplementary Table S5 Serum Urea, Crea, ALT, and AST Levels in Each Group of Mice （ $\bar{x}$ ± SD）

Group	Number	Urea（mmol/L）	Crea（μmol/L）	ALT（U/L）	AST（U/L）
NC	6	16.43±2.76	41.97±7.31	52.64±27.10	119.22±19.57

MC	6	15.09±2.23	44.34±9.31	71.29±20.74	154.55±14.39$^{\#}$
CTX	6	15.21±0.29	46.02±10.13	105.27±8.36*	184.12±6.98*
IFA-L	6	14.93±1.99	43.85±4.82	66.78±19.09$^{\triangle}$	156.07±21.61
IFA-M	6	14.19±1.92	41.98±5.09	71.66±25.78$^{\triangle}$	139.11±29.63$^{\triangle}$
IFAP-H	6	14.20±1.81	35.27±3.25	61.39±12.33$^{\triangle}$	128.10±28.30$^{\triangle}$

Note: Compared with the NC group, $^{\#}P<0.05$; Compared with the MC group, $^{*}P < 0.05$; Compared with the CTX group, $^{\triangle}P<0.05$.

Supplementary Table S6 Levels of TGF-β and TNF-α in Each Group of Mice（ $\bar{x}$ ± SD）

Group	Number	TNF-α（pg/mL）	TGF-β（pg/mL）
NC	6	519.58 ±26.45$^{*\triangle}$	111.41 ±24.26
MC	6	482.39±23.07$^{\#\triangle}$	126.37 ±5.10
CTX	6	585.53±31.72$^{\#*}$	127.28 ±23.97
IFA-L	6	511.27 ±23.26 $^{\triangle}$	103.78 ±4.17
IFA-M	6	520.58±18.16$^{*\triangle}$	74.64 ±6.48$^{\#*\triangle}$
IFA-H	6	619.04 ±26.92$^{\#*\triangle}$	67.77 ±6.56$^{\#*\triangle}$

Note: Compared with the NC group, $^{\#}P<0.05$; Compared with the MC group, $^{*}P < 0.05$; Compared with the CTX group, $^{\triangle}P<0.05$.
